# Supplementary material for: CRISPR-mediated correction of oncogenic AS-NMD in splicing factor mutant cancer
Source: Front Immunol. 2026 Oct 23;17:1958532. doi: 10.3389/fimmu.2026.1958532 (PMC13739707; doi:10.3389/fimmu.2026.1958532)
Supplement: Supplementary file 1 [file DataSheet1.pdf]

Supplementary Material

CRISPR-mediated Correction of Oncogenic AS-NMD in Splicing Factor Mutant Cancer

Preeti Nagar[1,3,†], Md Rafikul Islam[1,3,†], Nirjhor Anuvob Rahman[1], Shabiha Afroj Heeamoni[1], Md Mahbub Hasan[1], Saaimatul Huq[1], Fariha Alam Bushra[1], Mengye Hu[1], Rezwan Ali[2], Mokarram Hossain[2,3], Mohammad Alinoor Rahman[1,3,*]

[1]Department of Biochemistry and Molecular Biology, University of Arkansas for Medical Sciences, Little Rock, AR 72205, U.S.A.

[2]Department of Pharmaceutical Sciences, University of Arkansas for Medical Sciences, Little Rock, AR 72205, U.S.A.

[3]Winthrop P. Rockefeller Cancer Institute, University of Arkansas for Medical Sciences, Little Rock, AR 72205, U.S.A.

[†]These authors contributed equally to this work

[*]Address correspondence to:

Mohammad Alinoor Rahman (marahman@uams.edu)

Supplementary Table S1: Information of Cell Lines

Cell Lines	Source	Reference
HEK 293T	ATCC	N/A
MOLM-13	Omar Abdel-Wahab lab	Kim et al., Cancer Cell 2015
SET2	Omar Abdel-Wahab lab	Kim et al., Cancer Cell 2015
TF1	Omar Abdel-Wahab lab	Kim et al., Cancer Cell 2015
K562	ATCC (CCL-243)	N/A
$K562^{P95H}$	Omar Abdel-Wahab lab	Kim et al., Cancer Cell 2015
K052	AcceGen (ABC-TC0536)	N/A

Supplementary Table S2: Sequences of Primers

Primers	5' – 3' sequences
EZH2 RT-F	TTTCATGCAACACCCAACACT
EZH2 RT-R	CCCTGCTTCCCTATCACTGT
HPRT1-gDNA-F	GTCTTGCTCTGTTTCCCAG
HPRT1-gDNA-R	TTTAAAATATATTAGATAGCTCTAG
HPRT1 RT-F	AGGCGAACCTCTCGGCTTTC
HPRT1 RT-R	GAGGGCTACAATGTGATGGC
CDK5RAP2 RT-F	GCAGCTGCTCTCACAGAATG
CDK5RAP2 RT-R	CCTGGGAGGAATCAAACAGA
CRAT RT-F	AGCGAAGATGTTAGCCTTCG
CRAT RT-R	GCCTTCAGGTAGTGGTCCAG
SLC25A26 RT-F	CCGGAAGTTCAAGACAGACC
SLC25A26 RT-R	GAATCAGCATGCAAAAACCA
PFKM RT-F	CATCATCATTGTGGCTGAGG
PFKM RT-R	CACCTGGACACATTCCATGA
CDC45 RT-F	TTCCCGCCTATGAAGACATC
CDC45 RT-R	AAGCCAGCTCAAACATCACC
ATF2 RT-F	TTCTATGTACTGCGCCTGGA
ATF2 RT-R	GGTGTTGCAAGAGGGGATAA
MELK RT-F	ATGATCACCTCACGGCTACC
MELK RT-R	TGCAGGTGTTCTGCATAAGG
WDR45 RT-F	CAGGTGTGCGCATCTACAAC
WDR45 RT-R	ACAGAAAGCACTGGCTTGGT
DGUOK RT-F	CTTCGAGCACCCTTCAGTTC
DGUOK RT-R	GGGCTCCAGCTGTACTTTCA
ARMC10 RT-F	AACCTGAGTGTGAATGTTGAAAA
ARMC10 RT-R	GGGCACATTCTTCTCCATGT

Supplementary Table S3. Information of Antibodies

Antibody	Identifier	Source
EZH2	5246	Cell Signaling
GAPDH	2118	Cell Signaling
EED	MA5-55840	Thermo Fisher Scientific
SUZ12	3737	Cell Signaling
RBAP48	MA1-23273	Thermo Fisher Scientific
Tri-Methyl-Histone H3 (Lys27)	9733	Cell Signaling
Histone H3	4499	Cell Signaling

Supplementary Table S4. Sequences of pgRNA

pgRNA	5' – 3' sequences
pgHPRT1-S1-F	ACGCGTCTCACACCGCATACCTAATCATTATGCTGGTTTTAGAGCTAGAAATAGCAAGTT
pgHPRT1-S1-R	ACGCGTCTCAAAACCATGGACTAATTATGGACAGCGGTGTTTCGTCCTTTCCAC
pgHPRT1-S2-F	ACGCGTCTCACACCGTAAGAAAACAACAGGCCGGGGTTTTAGAGCTAGAAATAGCAAGTT
pgHPRT1-S2-R	ACGCGTCTCAAAACTCATGTGATTCAGCCCCAGTCGGTGTTTCGTCCTTTCCAC
pgEZH2-S1-F	ACGCGTCTCACACCGAGGAGTGGCTTGGTTCAAAGGTTTTAGAGCTAGAAATAGCAAGTT
pgEZH2-S1-R	ACGCGTCTCAAAACAATTGCTTAAAAATAGGATACGGTGTTTCGTCCTTTCCAC
pgEZH2-S2-F	ACGCGTCTCACACCGCAAAGTCTATGAGAGTTGCAGTTTTAGAGCTAGAAATAGCAAGTT
pgEZH2-S2-R	ACGCGTCTCAAAACGTCTTACTATTAAAACATCCCGGTGTTTCGTCCTTTCCAC
pgEZH2-S3-F	ACGCGTCTCACACCGAGCTATGGAAGAAACAGCATGTTTTAGAGCTAGAAATAGCAAGTT
pgEZH2-S3-R	ACGCGTCTCAAAACCTTCGTGTTAATATTTCAACCGGTGTTTCGTCCTTTCCAC

Supplementary Figure S1

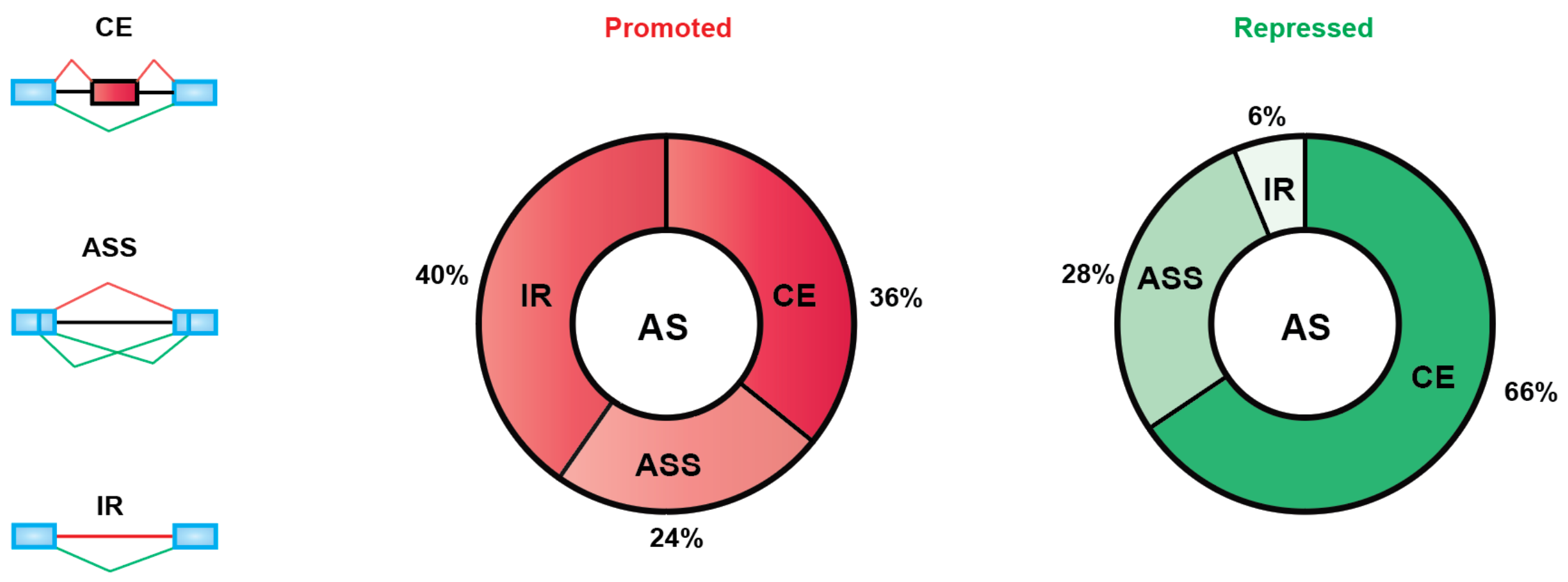

Supplementary Figure S1. Additional data related to Fig. 1. Proportion of differential splicing events (DSE) in TCGA-LAML RNA-seq of AML patients (SRSF2^{Mut} vs. SRSF2^{WT}) (Rahman et al. 2020b). ASS: alternative splice site selection; CE: cassette exon splicing; IR: intron retention.

Supplementary Figure S2

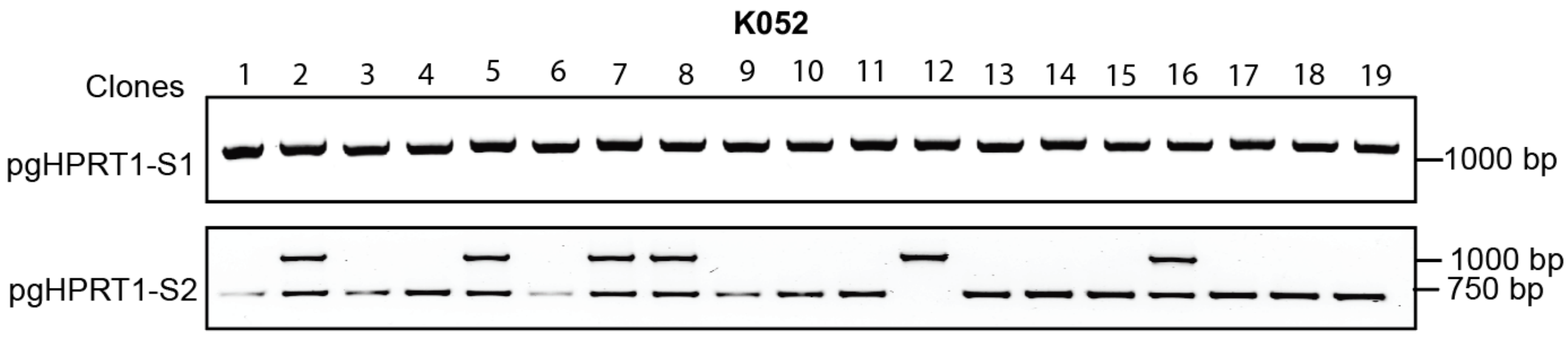

Supplementary Figure S2. Additional data related to Fig. 3. Genomic PCR of pgRNA-edited K052 clones targeting the *HPRT1* exon 2.

Supplementary Figure S3

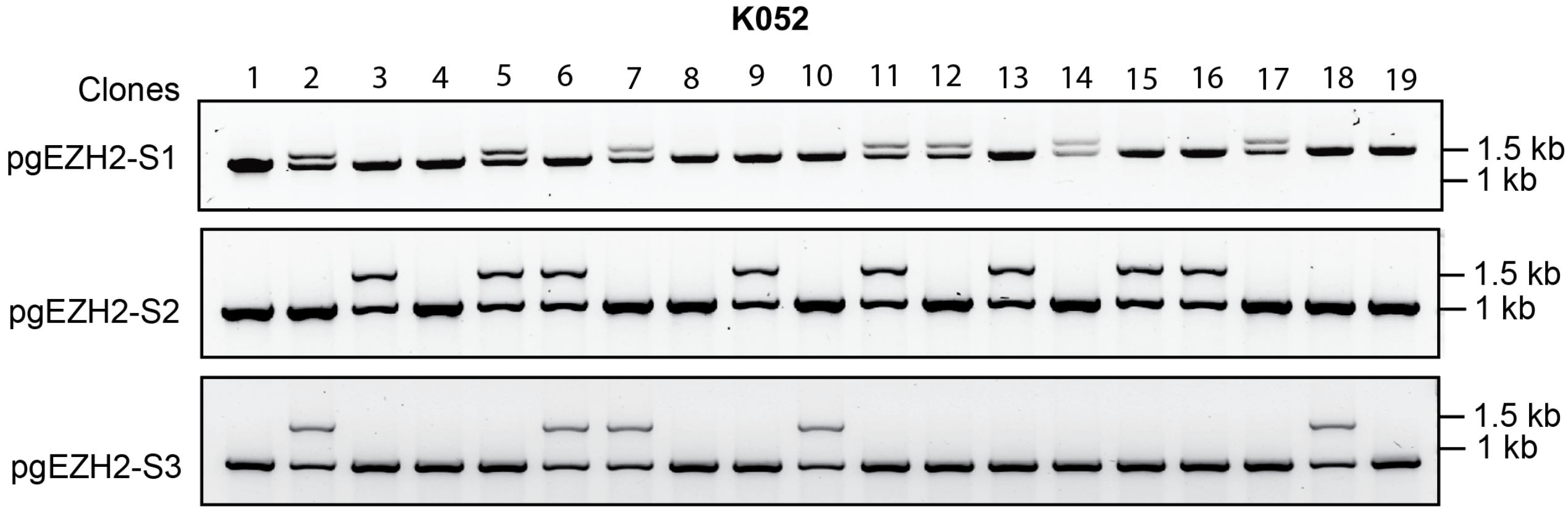

Supplementary Figure S3. Additional data related to Fig. 4. Genomic PCR of pgRNA-edited K052 clones targeting the *EZH2* PE.

Supplementary Figure S4

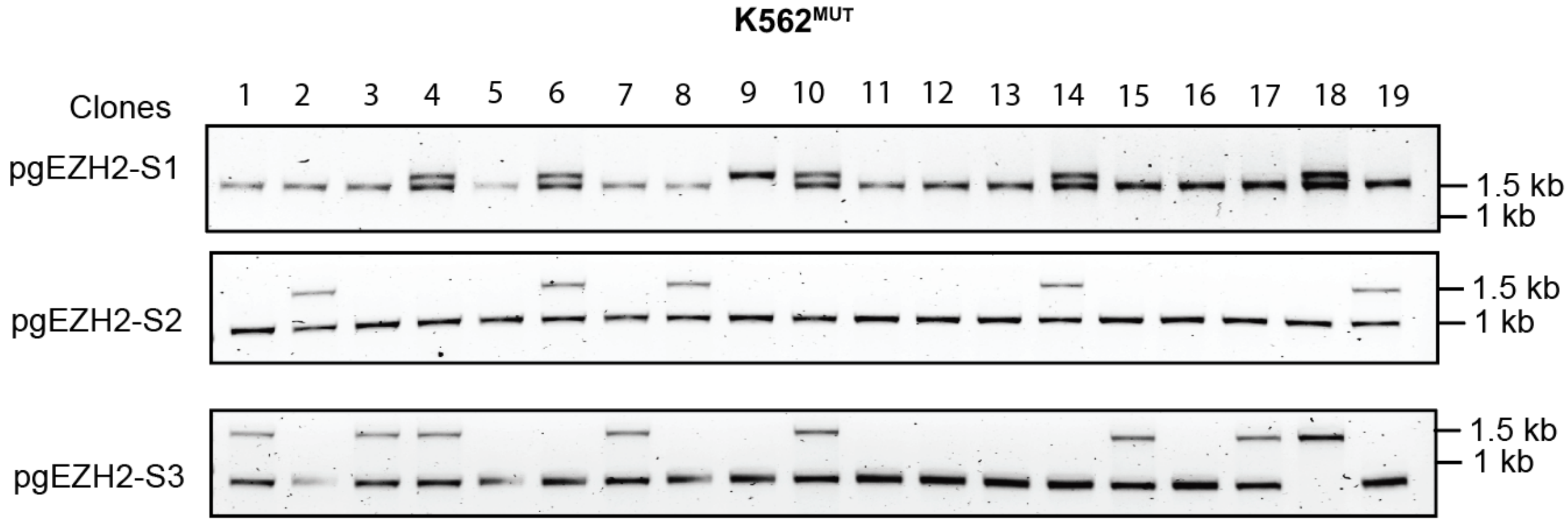

Supplementary Figure S4. Additional data related to Fig. 4. Genomic PCR of pgRNA-edited K562^{Mut} clones targeting the *EZH2* PE.

Supplementary Figure S5

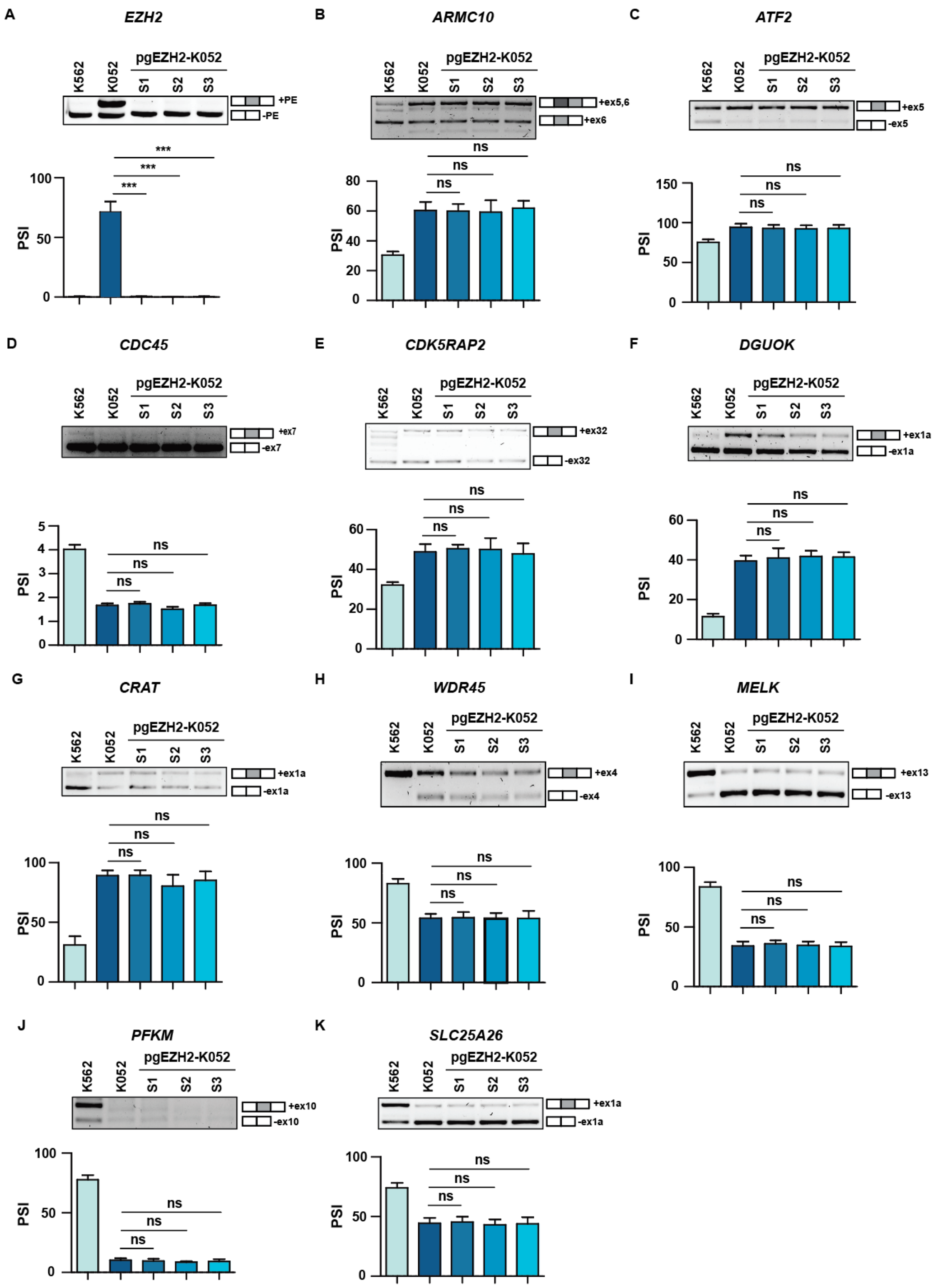

Supplementary Figure S5. Evaluating the on-target splicing modulation of the pgRNA-mediated exon deletion approach. **(A-K)** Top, representative RT-PCR gel showing alternative splicing profiles of the indicated endogenous genes in K562, K052 (control), and pgEZH2-edited K052 clones. Bottom, quantification of alternative splicing as percent spliced-in (PSI) is shown by a bar graph (mean ± SD, n=3 biological replicates using independent CRISPR clones, ***p<0.001, ns: not significant, one-way ANOVA with Tukey's multiple comparison test).

Supplementary Figure S6

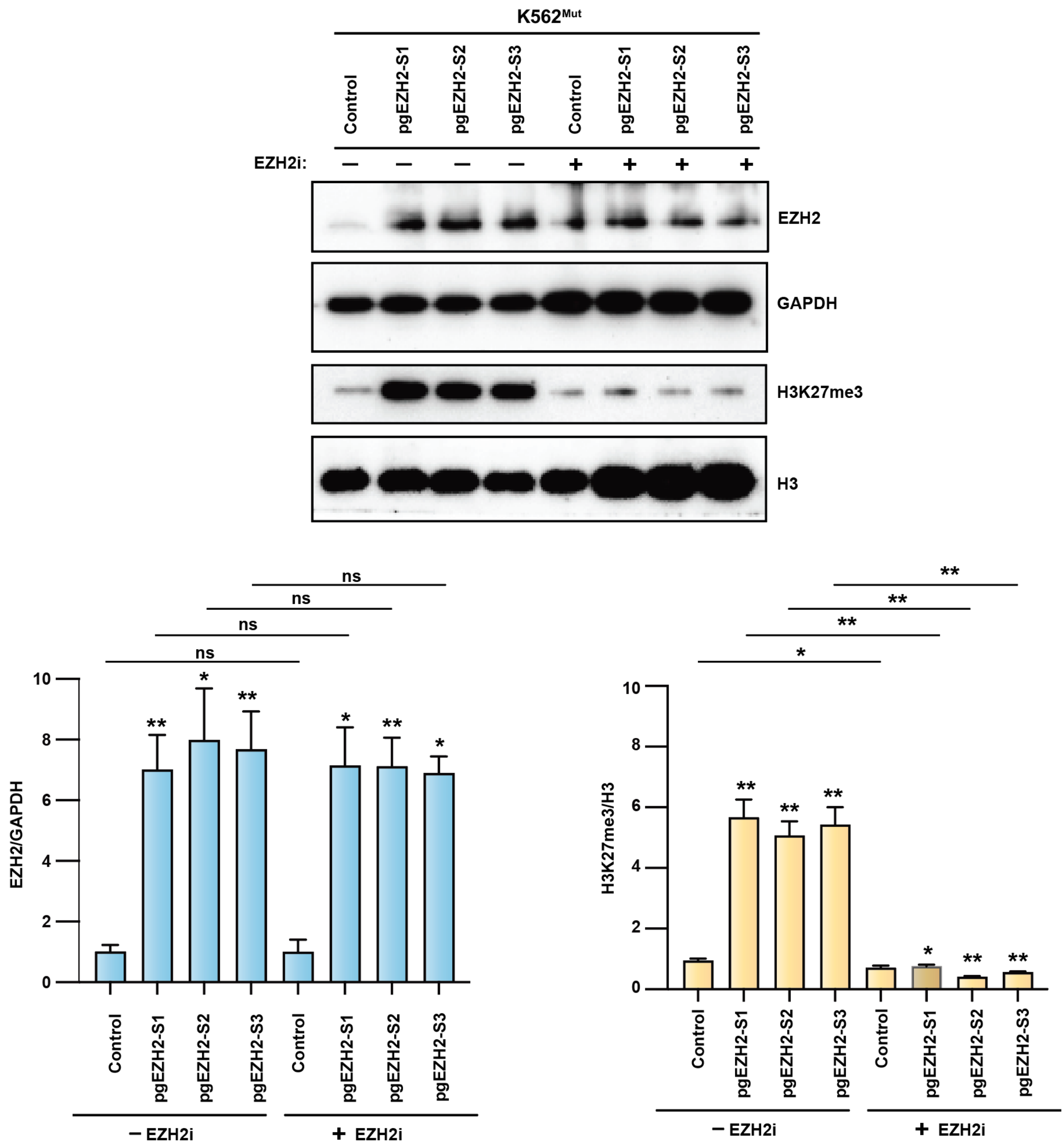

Supplementary Figure S6. Additional data related to Fig. 6. Top, representative western blotting of control (empty vector) or pgRNA-edited K562[Mut] clones treated without (-) or with (+) an EZH2 inhibitor (EZH2i) using the indicated antibodies. Bottom, quantification of the relative protein expression of EZH2 (EZH2/GAPDH) and H3K27me3 (H3K27me3/H3). Bar graph (mean ± SD, n=3 biological replicates using independent CRISPR clones, *p<0.05, **p<0.01, ***p<0.001, ns: not significant, two-way ANOVA with Tukey's multiple comparison test).
